# Epigenetic regulator MLL2 shows altered expression in cancer cell lines and tumors from human breast and colon

**DOI:** 10.1186/1475-2867-10-13

**Published:** 2010-04-30

**Authors:** Thanemozhi G Natarajan, Bhaskar V Kallakury, Christine E Sheehan, Margaret B Bartlett, Natarajan Ganesan, Anju Preet, Jeffrey S Ross, Kevin T FitzGerald

**Affiliations:** 1 Department of Oncology, Lombardi Comprehensive Cancer Center, Georgetown University Medical Center, 3970 Reservoir Road, Washington, DC-20057, USA; 2 Department of Pathology, Georgetown University Medical Center, Washington, DC-20057, USA; 3 Department of Pathology and Laboratory Medicine, Albany Medical College MC-81, 47 New Scotland Avenue, Albany, NY-12208, USA

## Abstract

**Background:**

MLL2, an epigenetic regulator in mammalian cells, mediates histone 3 lysine 4 tri-methylation (H3K4me3) through the formation of a multiprotein complex. MLL2 shares a high degree of structural similarity with MLL, which is frequently disrupted in leukemias via chromosomal translocations. However, this structural similarity is not accompanied by functional equivalence. In light of this difference, and previous reports on involvement of epigenetic regulators in malignancies, we investigated MLL2 expression in established cell lines from breast and colon tissues. We then investigated MLL2 in solid tumors of breast and colon by immunohistochemistry, and evaluated potential associations with established clinicopathologic variables.

**Results:**

We examined MLL2 at both transcript and protein levels in established cell lines from breast and colon cancers. Examination of these cell lines showed elevated levels of MLL2. Furthermore, we also identified incomplete proteolytic cleavage of MLL2 in the highly invasive tumor cell lines. To corroborate these results, we studied tumor tissues from patients by immunohistochemistry. Patient samples also revealed increased levels of MLL2 protein in invasive carcinomas of the breast and colon. In breast, cytoplasmic MLL2 was significantly increased in tumor tissues compared to adjacent benign epithelium (p < 0.05), and in colon, both nuclear and cytoplasmic immunostaining was significantly increased in tumor tissues compared to adjacent benign mucosa (p < 0.05).

**Conclusion:**

Our study indicates that elevated levels of MLL2 in the breast and colon cells are associated with malignancy in these tissues, in contrast to MLL involvement in haematopoietic cancer. In addition, both abnormal cellular localization of MLL2 and incomplete proteolytic processing may be associated with tumor growth/progression in breast and colonic tissues. This involvement of MLL2 in malignancy may be another example of the role of epigenetic regulators in cancer.

## Background

MLL2 (MLL) [Swiss-Prot: Q9UMN6] is a member of the MLL/trx family of proteins. It contains several evolutionarily conserved domains [1] including AT hooks at the N-terminus, cluster of PHD (plant homeodomain) zinc fingers associated with a bromodomain, and a SET (*s*uppressor of variegation, *e*nhancer of zeste, *t*rithorax) domain at the C-terminus [1]. The full length MLL2 (MLL2^FL^) is an uncleaved precursor protein with a predicted molecular weight of ~290 kD. MLL2^FL ^precursor protein undergoes post-translational proteolytic maturation, which is critical to its normal biological activity [2]. The enzyme responsible for MLL2 cleavage is taspase 1, and its consensus cleavage site (D/GVDD) is at a.a. 2063 [2]. Proteolytic cleavage generates a large N-terminus fragment with a predicted molecular weight of 215 kD, and a smaller C-terminus fragment which separates at ~75 kD in a denaturing gel. The cleaved fragments subsequently associate to generate a stable, functional, noncovalent heterodimeric complex [2].

The SET domain of MLL2 possesses histone H3 lysine 4 (H3K4) methyltransferase activity, and is an important component of the multi-protein complex involved in epigenetic gene regulation and embryonic development [3-5]. For example, *in vitro*, MLL2 complex has been shown to associate with Pax7, a transcription factor, and activate myogenic genes through H3 K4 methylation [4]. *In vivo*, Mll2 is shown to be required for normal embryonic development in mice [5-7]. A survey of the literature shows that several proteins with a primary function in epigenetic regulation and/or embryonic development are often aberrantly expressed in cancer. This finding is related to the observation that embryonic development and tumorigenesis share several common pathways [8]. Furthermore, proteins with chromatin remodeling motifs, such as PHD zinc fingers and SET domains, are often aberrantly expressed in tumors [9-11]. Considering all these features of MLL2, along with its significant structural similarity to MLL, we suspected that the *MLL2 *gene or its product may be altered in cancer, similar to it's paralog MLL, which is directly linked to haematopoietic tumorigenesis [12]. A literature survey, however, found only one published report describing *MLL2 *amplification through complex chromosomal rearrangements and duplications in human cancer cell lines [13]. Querying ONCOMINE, a publicly available source of gene expression data sets in cancers [14], we identified a few studies which listed *MLL2 *as one of the deregulated genes in some cancers-including melanoma, bladder and lung carcinomas-when compared to the corresponding normal tissues [14]. Subsequently, tissue microarray based preliminary screening in our laboratory also indicated that MLL2 may be disrupted in certain cancers. We, therefore, decided to investigate MLL2 expression in breast and colon cancer cell lines, and then substantiated our findings in archived formalin fixed paraffin embedded (FFPE) tumor tissues from patients with confirmed diagnoses of breast and colon cancers.

In order to study MLL2 in breast cancer cells, we selected a panel of six breast epithelial cell lines representing non-tumor breast epithelial derived cell lines (184A1 and MCF 10A) [15], weakly invasive breast tumor cell lines (T47D and MCF 7) [15,16] and highly invasive breast tumor cell lines (MDA-MB-157 and MDA-MB-231) [16]. For investigating MLL2 in colon cancer cells, we selected three cell lines derived from well-differentiated colon carcinomas (HT29, DLD-1 and Ls174T) [17-19] and three from poorly differentiated colon carcinomas (Lovo, Colo 205 and SW 480) [18,20,21]. We then substantiated our observations in cell lines by investigating MLL2 levels in breast and colon cancer tissues. Here we report that MLL2 expression is disrupted in invasive tumor cell lines and invasive carcinomas.

## Results

### MLL2 in breast cancer cell lines

#### MLL2 protein levels are elevated and MLL2 is incompletely processed in highly invasive breast cancer cell lines

**Figure 1:**
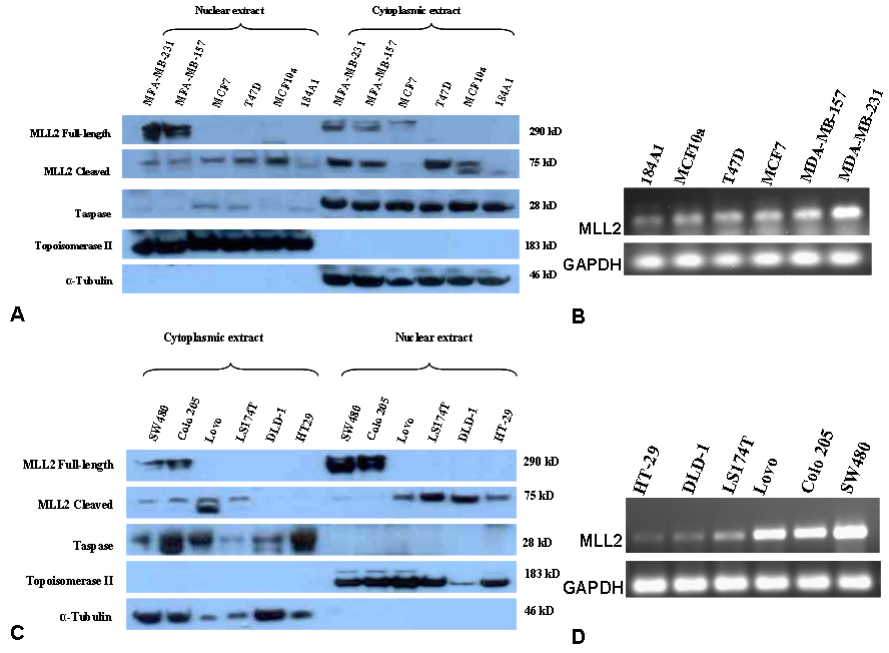
**Immunoblot images of nuclear cytoplasmic fractions, and gel images of reverse transcription-PCR (RT-PCR) from human mammary and colonic epithelial cell-lines**. (A & C) Cropped immunoblots of nuclear and cytoplasmic extracts from breast (A) and colon (C) cell lines probed for MLL2, taspase 1, topoisomerase IIβ (control nuclear protein) and alpha-tubulin (control cytoplasmic proteins). From top, full-length MLL2 (290 kD), cleaved C-terminus fragment of MLL2 (75 kD), topoisomerase IIβ and alpha-Tubulin are cropped images from the same blot. (B & D) Total RNA was isolated from mammary (B) and colonic (D) epithelial cell lines and subjected to RT-PCR using *MLL2 *and *GAPDH *sequence specific primers spanning exon-exon junctions. Cropped images of gels showing amplified products loaded on 2% agarose gels. Top- MLL2, bottom- GAPDH. A. Nuclear and cytoplasmic fractions from mammary epithelial cell lines from a 	single blot. B. MLL2 transcript from mammary epithelial cell lines from a single gel. C. Nuclear and cytoplasmic fractions from colonic epithelial cell lines from a single blot. D. MLL2 transcript from colonic epithelial cell lines from a single gel.

Human mammary cell lines--184A1, MCF10A, T47D, MCF7, MDA-MB-157 and MDA-MB-231--were processed to obtain nuclear and cytoplasmic extracts. Analysis of the extracts revealed the presence of a 75 kD fragment corresponding to the cleaved C-terminal segment of MLL2 in the nuclear and/or cytoplasmic fractions (Fig. 1A). The nuclear 75 kD signal was strongest in MCF10a and weakest in MDA-MB-231 and 184A1, while the cytoplasmic 75 kD signals were very intense in the more malignant cell lines (Table 1). Besides the 75 kD signal, in the cytoplasmic fractions of MCF7, MDA-MB-157 and MDA-MB-231, and the nuclear fractions of MDA-MB-157 and MDA-MB-231 cell lines, an additional signal was observed at ~290 kD, corresponding to the MLL2^FL ^(Table 1). The ~290 kD signal was either absent or faint in the nuclear and cytoplasmic fractions of 184A1, MCF10A and T47D (Fig 1A). Overall, MLL2 protein levels increased with increasing malignant potential of the cell lines.

**Table 1:** Relative levels of MLL2 protein in mammary epithelial cell lines

Cell lines	Origin, Description, ER status	Nuclear MLL2		Cytoplasmic MLL2	
		
		75 kD	290 kD	75 kD	290 kD
		
		(Cleaved)	(Uncleaved)	(Cleaved)	(Uncleaved)
**184A1**	Normal breast epithelium, immortalized, ER -	+	-	+	-

**MCF10a**	Normal breast epithelium, immortalized, ER-	+++	-	++	-

**T47D**	Early stage breast carcinoma, tumorigenic, non-metastatic, ER +	++	-	+++	-

**MCF7**	Early stage breast carcinoma, tumorigenic, non-metastatic, ER +	++	-	+	+ +

**MDA-MB-157**	Advanced breast carcinoma, Tumorigenic and metastatic, ER -	+	+ + ++	+++	+ + +

**MDA-MB-231**	Advanced breast carcinoma, Tumorigenic and metastatic, ER -	+	+ + ++	+++	+ + +

Distribution of cleaved and uncleaved MLL2 protein in nuclear and cytoplasmic fractions of normal and tumor derived mammary epithelial cell lines

**Staining intensity **- Negative; + Faint; **++ **Weak; **+++ **Moderate, ++++ Intense

#### MLL2 transcript levels are increased in cell lines derived from invasive tumors

Examination of MLL2 RNA levels by semi quantitative reverse transcription polymerase chain reaction (RT-PCR) in the mammary epithelial cell lines revealed highest levels of MLL2 transcript in MDA-MB-231 and least in 184A1 (Fig 1B). A gradual increase in the MLL2 RNA levels was observed in the order of 184A1, MCF10a, T47D, MCF7, MDA-MB-157 and MDA-MB-231.

#### Taspase 1 levels and its putative MLL2 cleavage site are not altered

Examination of endogenous taspase 1 (enzyme responsible for proteolytic processing of MLL2) protein levels, revealed bands of consistent intensities in the cytoplasmic fractions of the breast cell lines used in this study (Fig 1A). Additionally, analyses of the *MLL2 *sequences coding for the taspase 1 cleavage site showed no alterations in cell lines carrying MLL2^FL ^(data not shown).

### MLL2 in colon cancer cell lines

#### MLL2 protein is elevated and incompletely processed in poorly differentiated colon cancer cell lines

Established cell lines from colon cancer tissues--HT29, DLD-1, Ls174T, Lovo, Colo205 and SW480--were processed to obtain nuclear and cytoplasmic extracts. Analysis of these nuclear-cytoplasmic extracts revealed a 75 kD signal corresponding to the cleaved, C-terminal peptide of MLL2 in the nuclear and/or cytoplasmic fractions (Fig 1C, Table 2). The 75 kD band was most intense in the nuclear fractions of HT-29, DLD-1, Ls174T and Lovo, and least in Colo205 and SW480. In the cytoplasmic fractions, the cleaved MLL2 appeared in all cell lines except HT29 and DLD-1. Additionally, Colo205 and SW480 displayed abundant levels of uncleaved MLL2^FL ^(Table 2) in nuclear fractions compared to the cytoplasmic fractions (Fig 1C).

**Table 2:** Relative levels of MLL2 protein in colonic cell lines

Cell lines	Nuclear MLL2		Cytoplasmic MLL2	
	
	75 kD	290 kD	75 kD	290 kD
	
	(Cleaved)	(Uncleaved)	(Cleaved)	(Uncleaved)
**HT29**	+	-	-	-

**DLD-1**	++	-	-	-

**Ls174T**	++	-	+	-

**Lovo**	+	-	++	-

**Colo205**	-	+ + ++	+	+ +

**SW480**	-	+ + ++	+	+ +

Distribution of cleaved and uncleaved MLL2 protein in nuclear and cytoplasmic fractions of colon carcinoma cell lines

**Staining intensity **- Negative; + Weak; **+++ **Moderate, ++++ Intense

#### MLL2 RNA levels are increased in cell lines derived from poorly differentiated colonic tumors

MLL2 RNA levels were found to be higher in the colon cell lines -- Lovo, Colo205 and SW480-compared to HT-29, DLD-1 and Ls174T (Fig 1D).

#### Taspase 1 levels and putative cleavage sites are not altered in colon cancer cell lines

Immunoblots of the cell line derived fractions were probed with anti-taspase 1 antibody, which revealed variable amounts of taspase 1 in the cytoplasmic fractions of the colonic cell lines examined (Fig 1C). DNA sequence analyses using primers flanking the coding sites for taspase 1 cleavage site in the *MLL2 *gene revealed no alterations in the colonic cancer cell lines carrying MLL2^FL ^(data not shown).

### MLL2 expression measured by immunohistochemistry, in breast tumor and normal adjacent tissues

#### Patient demography

The results presented in this study originated from ninety-six female patients with breast carcinoma, ranging in age from 26 to 89 years, with a mean age of 59 years at the time of diagnosis. Lymph node (LN) status was available for 95 (99%) cases. Tumor size was available for 90 (94%) cases and tumor grades were available for 68 (71%) cases. Estrogen receptor (ER) and progesterone receptor (PR) status was known for 88 (92%) and 77 (80%) cases, respectively. HER-2/neu status was available for 87 (91%) cases. Overall survival status was known for all, and recurrence data was available for 95 (99%) cases.

Tumor and benign tissues were present in all (100%) cases with invasive carcinoma (IC) of the breast. Sixty-three (66%) were ductal and 33 (34%) were lobular carcinomas. LN status was positive in 50/95 (55%), and negative in 41/95 (45%) cases. Tumor size was ≤ 2 cm for 36/90 (40%), and >2 cm for 54/90 (60%) cases. Of the sixty-eight graded tumors, 18 (26%) were well differentiated, 33 (49%) moderately differentiated, and 17 (25%) poorly differentiated. There were 81/96 (84%) early stage (1 and 2), and 15/96 (16%) advanced stage (3 and 4) tumors. ER was positive in 64/88 (73%) cases while PR in 43/77 (56%) cases, and 21/87 (24%) cases were HER2/neu positive. Thirty-seven (38%) cases had post-surgical disease recurrence, and 59/96 (62%) had expired.

#### Immunohistochemically detected MLL2 expression was significantly elevated in tumor tissues compared to adjacent benign tissue

**Figure 2:**
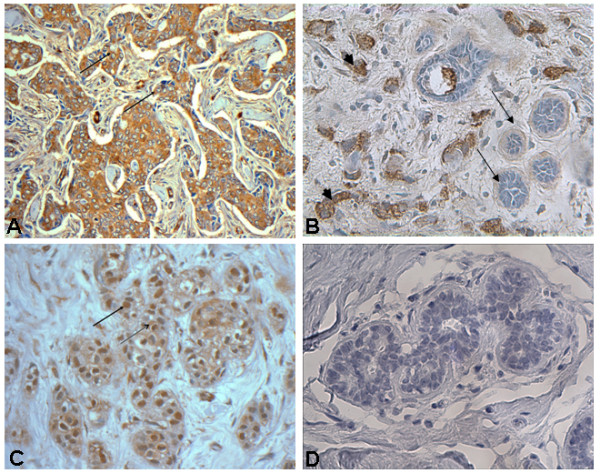
**Representative immunohistochemistry staining for MLL2 in human FFPE breast tissue sections **A. Intense cytoplasmic MLL2 immunostaining in epithelial cells (arrows) of tissue section from invasive breast carcinoma, ×400. B. Breast tissue showing strong cytoplasmic immunoreactivity of MLL2 in invasive ductal carcinoma (solid arrow head) cells compared to the adjacent, benign (arrows) mammary ducts, ×200. C. Strongly positive nuclear (arrow) MLL2 immunostaining in epithelial cells of invasive breast carcinoma tissue section, ×400. D. Negative control, ×400.

Cytoplasmic MLL2 immunostaining was increased (Fig. 2A, Table 3) in the malignant tissues in 86/96 (90%) cases (p < 0.05) compared to benign epithelium from the same patients (Fig 2B). Cytoplasmic expression correlated with early tumor stage (p = 0.02), and menopausal status (p = 0.03) (Table 4). Nuclear immunoreactivity was observed in 43/96 (45%) cases (Fig. 2C) and did not correlate with any of the clinicopathologic variables. Distribution pattern for nuclear immunoreactivity is detailed in Table 3.

**Table 3:** Immunohistochemically detected MLL2 expression in human breast tissue (n = 96)

	Nuclear MLL2 staining	Cytoplasmic MLL2 staining
**Positive staining**	43/96 (45%)	96/96 (100%)

**Tumor vs. Benign**		

T < B	17/43 (39%)	1/96 (1%)

T = B	8/43 (19%)	9/96 (9%)

T > B	18/43 (42%)	86/96 (90%)*

Distribution of breast cancer cases based on nuclear and cytoplasmic MLL2 immunostaining in tumor versus benign tissues; *p < 0.05

**Table 4:** MLL2 expression and clinicopathological variables in breast cancer (n = 96)

	Number of cases positive for cytoplasmic MLL2	Number of positive cases where T > B (%)
**N**	**96**	86 (90)

**Tumor stage**		

Early (1 & 2)	81	75 (93)*

Advanced (3 & 4)	15	11 (73)

**Menopausal status**		

Pre	20	16 (80)

Post	76	70 (92)**

Clinicopathological variables associated with elevated levels of cytoplasmic MLL2 immunoreactivity in patients with invasive breast cancer; *p = 0.02; **p = 0.03

### MLL2 expression by immunohistochemistry, in tumor and adjacent normal tissues of the colon

#### Patient demography

Sixty-six patients, including 34 (52%) males and 32 (48%) females, who had invasive carcinoma of the colon were included in this study. The patients ranged in age from 38 to 92 years, with a mean age of 70 years at diagnosis. Tumor and benign tissue were present in all 66 cases with IC of the colon. LN status was positive in 32/66 (49%) cases, and negative in 34/66 (51%) cases. There were 4/66 (6%) well differentiated, 54/66 (82%) moderately differentiated, and 8/66 (12%) poorly differentiated tumors. There were 18/66 (27%) early stage (1 and 2), and 48/66 (73%) advanced stage (3 and 4) tumors. Survival status was available for all cases and 50/66 (76%) patients had expired.

#### Immunohistochemically detected MLL2 expression is significantly elevated in tumor tissues compared to adjacent benign mucosa

**Figure 3:**
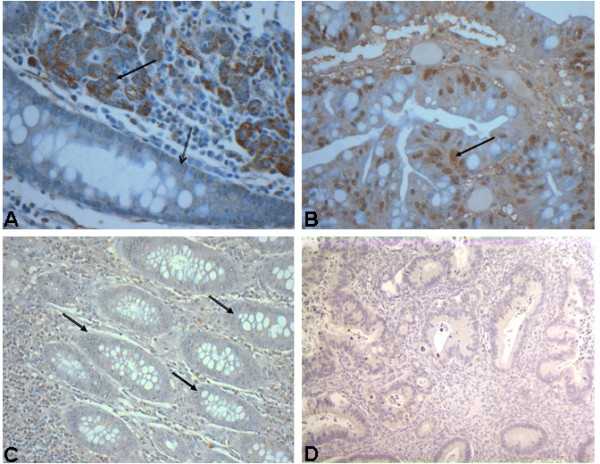
**Representative immunohistochemistry staining for MLL2 in human FFPE colonic tissue sections**. A. Note differential cytoplasmic MLL2 immunostaining in tissue section from invasive carcinoma of the colon (solid arrow) and adjacent normal mucosa (hollow arrow), ×400. B. Nuclear immunoreactivity of MLL2 (arrow) in invasive colon carcinoma, ×400 C. Weakly positive MLL2 immunostaining in adjacent benign colonic epithelial cells (arrows), ×200 from same patient whose tumor sample is shown in (B) D. Anti-MLL2 antibody was incubated with blocking peptide and added to the slide with FFPE colon tissue, followed by washing and staining with secondary antibody and visualization by using diaminobenzidine (DAB) as a precipitating enzyme product.

Cytoplasmic MLL2 immunostaining was significantly increased in 63/66 (95%) tumors (p < 0.05) when compared to adjacent benign mucosa (Fig 3A, Table 5). No correlation was observed between cytoplasmic MLL2 expression and any of the clinicopathologic variables. Nuclear staining was increased (Fig 3B) in 77% cases (p < 0.05) (Table 5) compared to benign mucosa from the same cases (Fig 3C). Increased nuclear MLL2 expression correlated with early stage tumors (p = 0.03) (Table 6) and absence of LN involvement (p = 0.005) (Table 6).

**Table 5:** Immunohistochemically detected MLL2 expression in human colonic tissues

	Nuclear MLL2 staining	Cytoplasmic MLL2 staining
**Positive cases**	35/66 (53%)	66/66 (100%)

**Tumor vs. Benign**		

T < B	7/35 (20%)	3/66 (5%)

T = B	1/35 (3%)	0/66 (0%)

T > B	27/35 (77%)*	63/66 (95%)*

Distribution of colon cancer cases based on nuclear and cytoplasmic MLL2 immunostaining in tumor versus benign tissue; * p < 0.05

**Table 6:** MLL2 expression and clinicopathological variables in breast cancer (n = 96)

	Number of cases positive for nuclear MLL2	Number of positive cases where T > B (%)
	**35**	**27 (77)**

**Lymph node**		

Negative	20	19 (95)***

Positive	15	8 (53)

**Tumor stage**		

Early	12	11 (92)**

Advanced	23	16 (70)

Clinicopathological variables associated with elevated levels of nuclear MLL2 immunoreactivity in patients with invasive colon carcinoma; **p = 0.03; ***p = 0.005

## Discussion

In the present study we observed that: (a) MLL2, primarily regarded as a nuclear protein with nuclear localization signals, displayed significant cytoplasmic localization in both normal and malignant cells, (b) mammary and colonic cell lines derived from highly invasive tumors exhibited altered sub-cellular distribution and proteolytic processing of MLL2 compared to non-tumor/less-invasive-tumor cell lines, and (c) MLL2 is overexpressed in breast and colon tumors tissues compared to the corresponding normal adjacent tissues.

MLL2 is primarily regarded as a nuclear protein. However, we observed both nuclear and cytoplasmic MLL2 in cell lines and tissue sections from breast and colon. In the breast epithelial cell lines, MLL2 specific bands representing the cleaved (75 kD) protein were observed in the cytoplasmic fractions of not only the tumor cell lines but also the non-tumor cell lines, 184A1 and MCF10a (Fig 1A); while bands representing the full-length, unprocessed MLL2 (290 kD) were present in the highly invasive tumor cell lines alone, and which may be related to oncogenic activity. The nuclear-cytoplasmic localization pattern of MLL2 was also evident in the benign sections of both breast and colonic tissues, but it was not possible to delineate the cleaved from the uncleaved MLL2 as the antibody used in this study cannot differentiate one from the other *in situ *(details of antibody specificity are described in Methods section). Considering this cytoplasmic presence of MLL2 in the non-tumor cell lines and non-tumor tissue sections, it is possible that MLL2 may have a yet unidentified function in the cytoplasm, besides its role in epigenetic regulation in the nucleus [22,23]. However, a notably increased cytoplasmic presence in the cancer cell lines and cancer tissues, together with the presence of MLL2^FL ^could be a tumor-related anomaly resulting from overexpression or increased protein/RNA stability.

Our study of the breast and colonic cell lines revealed two notable trends related to the proteolytic processing of MLL2. First, we observed a gradient in the intensities of the 75 kD signal in the nuclear fractions of both breast and colonic cell lines (Fig. 1A &1C). In the breast epithelial cell lines, the intensities of the 75 kD nuclear MLL2, indicative of normal proteolytic processing, showed a decreasing trend with increasing malignancy from MCF10A to MDA-MB-231 except for MCF7. Of these cell lines, 184A1 and MCF10A are non-tumorigenic, MCF7 [16] and T47D are tumorigenic [15] but weakly invasive [24], while MDA-MB-157 and MDA-MB-231 [16] are highly invasive [15,24,25]. The non-tumor cell line, 184A1, did not fit this trend and this discrepancy could be attributed to an overall lower level of MLL2 in 184A1, and/or the difference in proliferation rate--184A1 cells are reported to have a relatively lower proliferation rate compared to MCF10A [25]. In the colonic epithelial cell lines a similar intensity gradient was noted for the 75 kD nuclear MLL2 fragment. Of these cell lines, HT29 [17], DLD-1 [18] and Ls174T [19] are derived from well-differentiated tumors while, Lovo [18], Colo205 [20] and SW480 [21] are derived from poorly differentiated tumors; and the 75 kD nuclear MLL2 was least in the Colo205 and SW480 cell lines.

The second notable trend was the presence of an additional band of 290 kD size (corresponding to the uncleaved precursor MLL2^FL^) in the more invasive/poorly differentiated cell lines. More importantly, the MLL2^FL ^(290 kD) signal intensity increased in the nuclear and cytoplasmic fractions as the 75 kD nuclear signal decreased (Fig. 1A &1C). This uncleaved MLL2^FL ^observed in the cytoplasmic and nuclear fractions of the advanced tumor cell lines could be a consequence of insufficient endogenous taspase 1 required for processing the excess MLL2. Since proteolytic cleavage could also be impaired as a consequence of non-cleavable mutations in MLL2^FL ^or the absence/decrease in the endogenous taspase 1 that cleaves MLL2 [2], we looked for mutations in the *MLL2 *sequence coding for taspase 1 cleavage site, and also examined taspase 1 protein levels in the cell lines. Sequence analysis of the coding region for taspase 1 cleavage sites in cell lines carrying MLL2^FL ^did not reveal any alterations in the cleavage site encoding sequences. In addition, we found that the taspase 1 protein levels did not vary in parallel with the presence or absence of MLL2^FL^. Taspase 1 protein levels were consistent across the six breast tissue cell lines, irrespective of the presence of MLL2^FL^. Although we did not observe consistent levels of taspase 1 across the colonic cell lines, we did observe that the presence or absence of MLL2^FL ^and its levels, failed to correlate with taspase 1 level. That is, higher levels of taspase 1 did not correspondingly correlate with decreased levels of MLL2^FL ^or its total absence. These results suggest that the presence of MLL2^FL ^in the invasive cell lines is not a consequence of diminished levels of taspase 1 or a mutated cleavage site in the *MLL2*. Further investigation is required, to determine the cause and consequences of MLL2^FL ^in the nucleus, which might be related to the shift in the nuclear-cytoplasmic localization of MLL2 in the invasive cell lines. Whatever may be the cause, our results suggest that the presence of precursor MLL2^FL ^is associated with a higher degree of malignancy.

According to an earlier report [2], proteolytic processing of MLL2^FL ^is crucial to its stability, sub-nuclear localization, and methyltransferase activity. Impaired proteolytic maturation could result in significant changes in the normal epigenetic regulatory activities of MLL2. It has been shown *in vitro *that MLL2 forms a multiprotein complex with Wdr5-Ash2L [26] and associates with proteins like Pax7 and NF-E2 to direct histone lysine methylation at specific gene loci [3,4]. MLL2 specific histone methylation complex is also known to associate with the tumor suppressor protein, menin, and mediate histone methylation at *Hoxc8 *locus [27]. Given the critical role for MLL2 in histone methylation activities, we believe that proteolytically immature and/or inappropriately expressed MLL2 may fail to effectively associate with the other members of the histone-methyltransferase complex, which in turn can adversely affect its role in epigenetic gene regulation.

In our analysis of the breast and colonic cell lines by reverse transcription (RT)-PCR, we also observed that MLL2 RNA levels were highest in the invasive tumor cell lines and least in the non-tumor/less-invasive-tumor cell lines (Fig. 1B &1D). This trend in the MLL2 RNA levels was consistent with our observation of the overall increase in protein levels in the invasive tumor cell lines and in the tumor tissues. Since a real-time measurement was not performed on these cell lines it is not known if the increased levels of RNA resulted from an increased rate of transcription. The observed gradient in RNA levels in the breast and colon cell lines could also be due to differences in RNA stability. Though the RT-PCR results are not strictly quantitative, the results do indicate that MLL2 RNA levels are more abundant in the highly invasive/less differentiated cell lines.

Finally, our study on tissue sections from breast and colon cancer patients revealed that immunohistochemically detected MLL2 is significantly increased in tumor tissues. In breast tumors from patient samples, cytoplasmic MLL2 was significantly overexpressed as compared to normal adjacent tissues, and in colon tumors both cytoplasmic and nuclear MLL2 were significantly overexpressed when compared to adjacent benign mucosa. Since immunohistochemical signals can often arise from non-specific antibody reactions, we evaluated the specificity of antibody reactivity using a blocking peptide, a part of which represented the epitope recognized by anti-MLL2 antibody (detailed in Methods section). The peptide blocks the ability of the antibody to bind to its antigen. These experiments confirmed a high specificity of the antibody to the MLL2 antigen (Fig. 3D).

Analysis of MLL2 expression data with clinicopathological variables revealed a small correlation between MLL2 overexpression and early tumor stages (breast and colon) and absence of lymph node involvement (colon). However, the number of cases in each category was too small and the association too tentative to draw a substantive conclusion at this juncture. Despite a lack of correlation with established clinicopathological variables, the elevated levels of MLL2 protein in both breast and colon cancer was significant. These results are in line with our observation of increased MLL2 protein and RNA levels in the cell lines. However, due to the lack of an appropriate antibody to distinguish the cleaved MLL2 from uncleaved MLL2^FL ^by immunohistochemistry, we cannot comment on the composition of the overexpressed protein as to whether it constituted more of the 75 kD fragment or the 290 kD MLL2^FL^. The elevated levels of MLL2 in the tumor tissues could be the result of overexpression, genomic amplification, increased RNA and/or protein stability, or, at least in part, due to alterations in protein processing. For example, genomic amplification of *MLL2 *(through complex chromosomal rearrangements or chromosomal duplications) resulting in four *MLL2 *copies has been previously reported in one of the breast cancer cell line, MDA-MB-157, used in this study [13].

Whichever may be the cause, deregulated expression of MLL2 and/or defective proteolytic processing may adversely impact MLL2 mediated histone methylation activities, and in turn, disrupt downstream target genes potentially involved in cell cycle or cell proliferation activities. Consequently, aberrantly expressed MLL2 driven epigenetic regulation may contribute to tumor growth and/or progression. If such is the case, deregulation of MLL2 may be a more generic feature in tumorigenesis rather than an event specific to a particular tumor type, as is indicated by our findings in both breast and colon tumors.

## Conclusions

In all, our study shows for the first time that overexpression of MLL2, an altered subcellular distribution, and aberrant proteolytic processing may be linked to tumorigenesis in mammary and colonic tissues. This linkage may be attributed, at least in part, to the role of MLL2 in histone methyltransferase activities [22,23]. Given the well-known role for MLL in leukemias, and the increasing focus on abnormal epigenetic regulation in cancer, the results of this study present a strong argument for further investigation of MLL2 and its possible involvement in solid tumors.

## Methods

### Cell lines, nuclear-cytoplasmic fractionation, immunoblotting

#### Cell lines

All parental cell lines were initially purchased from American Type Culture Collection (ATCC) and maintained at the University's Tissue Culture Core facility. Human breast epithelial cell lines used in this study included 184A1, MCF10A, T47D, MCF7, MDA-MB-157 and MDA-MB-231. MCF10A and 184A1, are immortalized human mammary epithelial cell lines derived from non-tumor breast tissue [15,16]. T47D and MCF7 are ER+, tumorigenic, non-metastatic, anti-estrogen sensitive cell lines. MDA-MB-157 and MDA-MB-231 are ER-, tumorigenic, metastatic and anti-estrogen resistant cell line.

The colonic epithelial cell lines used in this study included HT29, DLD-1, Ls174T, Lovo, Colo205 and SW480, and were initially obtained from ATCC and maintained at the Tissue Culture Core facility of the University. HT29 [17], DLD1 [18] and Ls174T [19] were established from well differentiated carcinomas while Lovo [18], Colo205 [20] and SW480 [21] were derived from poorly differentiated colon carcinomas.

Cell lines were maintained at 37°C, in a humidified chamber with 5% CO_2_, in T-175 cm^2 ^tissue culture flasks. MCF10A was grown in a 1:1 mixture of Ham's-F12 medium and DMEM (Gibco, USA), with 2.5 mM L-glutamine, and supplemented with 20 ng/ml epidermal growth factor, 0.01 mg/ml insulin, 500 ng/ml hydrocortisone, and 5% horse serum. Cell line 184A1, was grown in MEGM^® ^(Clonetics^®^) complete media (10 ng/ml hEGF, 5 ug/ml insulin, 0/5 ug/ml hydrocortisone and bovine pituitary extract). All other breast cell lines were cultured in DMEM complete media containing 4.5 g/L glucose, 2 mM L-glutamine, 1 mM sodium pyruvate, 0.1 mM non-essential amino-acids, and supplemented with 5% fetal bovine serum (FBS). The colonic epithelial cell lines HT-29, Ls174T and SW480 were grown in DMEM complete media. DLD-1 and Colo205 were cultured in RPMI 1640 media supplemented with 10% FBS, 1% Sodium pyruvate and 1% non-essential amino acids. Lovo was cultured in Ham's F12K media supplemented with 10% FBS.

#### Nuclear-Cytoplasmic fractionation

Equal volumes of the cell pellet from each cell line were used for fractionation following an established protocol [28]. Known volumes of cell pellets were resuspended in a hypotonic buffer (HB) (10 mM Tris pH 7.9, 1.5 mM MgCl_2_, 10 mM KCl, 20% TritonX-100 and protease inhibitors (Roche, Germany)), and incubated on ice for 30 minutes with vortexing for 30 s at intervals of 10 minutes. The suspension was then centrifuged at 1000 g, pellets were saved, and the supernatant was centrifuged at 7000 r.p.m. in a fresh tube to remove debris. To the supernatant, 5 M NaCl was added to achieve a final concentration of 200 mM cytoplasmic extract. The saved pellet was resuspended in high salt concentration buffer C (10 mM Tris pH 7.9, 1.5 mM MgCl2, 10 mM KCl, 400 mM NaCl, 0.4% TritonX-100 and protease inhibitors) and vortexed for 30 minutes at 4°C, followed by centrifugation at 20,000 g. The supernatant was transferred to a fresh tube and an equal volume of HB was added to achieve a final concentration of 200 mM NaCl. This constituted the nuclear extract.

#### Immunoblotting and antibodies

Extracts were separated on a 3-8% Tris-acetate denaturing gel (NuPAGE, Invitrogen, USA), and blotted onto a PVDF membrane. The blots were probed with anti-MLL2 (see Immunohistochemistry section for details) antibody (1:500) in 5% non-fat dry milk dissolved in tris-buffered saline with 0.1% Tween-20, followed by goat anti-rabbit peroxidase conjugated secondary antibody (Santa Cruz Biotechnology, USA). The blots were subsequently probed with anti-taspase 1 (Santa Cruz Biotechnology, USA) (1:200) followed by secondary antibody. As evidence of purity of the nuclear and cytoplasmic fractions, blots were probed with mouse monoclonal anti-Topoisomerase IIβ (1:1000) (BD Biosciences, USA) and anti-alpha-tubulin (1:5000) (Sigma, USA), respectively. The signals were detected by incubating the membrane with ECL chemiluminescent substrate (Immobilon substrate, Millipore, USA) for 5 minutes and then exposing an X-ray film (Pierce, USA) for 30 s.

### DNA isolation and sequencing

DNA from cell lines were isolated and purified by spin column (Epicenter^® ^Biotechnologies, WI, USA). PCR was performed with primers designed to amplify genomic DNA sequences coding for the putative cleavage site for taspase 1. The primer sequences were- forward 5'-*caggactgagtgctgctgac*-3' and reverse 5'-*agtatgattttggatgtggcgggt-*3'. Amplified PCR products were purified by spin columns and sequenced at the University's core facility.

### RNA isolation and semi-quantitative reverse transcription-PCR

Total RNA was extracted from cell lines using QIAzol lysis reagent (Qiagen, USA). Primers were designed spanning adjacent exon-exon junctions. Primer sequences were- forward *5'-tctcacggtgccaagatgg -3' *and reverse *5'-tcggggcgctcgacctcgct -3'*. GAPDH (control) primer sequences were- forward *5'-tgcaccaccaactgcttagc-3' *and reverse *5'-ggcatggactgtggtcatgag-3'*. To determine transcript levels of MLL2, one-step reverse transcriptase-PCR (RT-PCR) was performed using equal amounts of total RNA from each cell line. Amplified products were separated on a 2% agarose gel containing ethidium bromide. Gels were imaged using FluorChem™ IS-8900 imaging system, CA, USA (Alpha Innotech).

### Patients and Clinicopathology

#### Mammary Carcinoma

Formalin fixed paraffin embedded (FFPE) tissue sections from 96 patients who underwent either mastectomy or local excision for primary invasive mammary carcinoma, between 1983 and 1997, at the Albany Medical Center Hospital were randomly selected for the current study. This study was approved by the Albany Medical Center Institutional Review Board (IRB). The pathology and clinical records, tissue blocks, and hematoxylin and eosin stained slides were retrieved for each case. Slides were reviewed and samples were identified based upon the presence of adequate tumor tissue, the representative nature of the overall grade, and the presence of adjacent normal (non-tumor) epithelium. Tumor type, age at diagnosis, lymph node (LN) status, tumor size, tumor grade, pathologic stage, estrogen and progesterone steroid hormone receptor status, HER-2/neu status, recurrence, and overall survival were obtained by review of the medical records. Menopausal status was considered by defining premenopausal age as ≤ 55 years, and postmenopausal age as >55 years. Tumors were graded using the modified Bloom and Richardson method [29]. This method is also the accepted standard for grading lobular carcinomas [29]. All cases were staged according to American Joint Committee on Cancer [30] criteria, using the TNM classification scheme, at the time of diagnosis. Estrogen/progesterone receptor (ER/PR) status was measured by competitive binding assays and immunohistochemistry (IHC). HER-2/neu protein status was detected by immunohistochemistry (IHC), or HER-2/*neu *gene amplification status measured by fluorescence in situ hybridization (FISH), or both.

#### Colorectal Carcinoma

FFPE tissue sections from 66 patients who underwent surgery for primary invasive colorectal carcinoma, between 1990 and 2000, at the Albany Medical Center Hospital, were randomly selected for the current study. This study was approved by the Albany Medical Center IRB. The pathology and clinical records, tissue blocks, and hematoxylin and eosin stained slides were retrieved for each case. Slides were reviewed and samples were identified based upon the presence of adequate tumor tissue, the representative nature of the overall grade, and the presence of adjacent benign epithelium. LN status was recorded for all cases. All tumors were graded and staged according to the Duke's system [31].

### Immunohistochemistry

#### MLL2 Immunostaining

The anti-MLL2 polyclonal antibody [27] was purchased from Bethyl Labs, Inc., MD, USA (Cat # A300-113A). This antibody is specific to an epitopic region upstream of the SET domain at the C-terminus, between residues 2375-2425. The antibody targets uncleaved MLL2^FL ^as well as cleaved fragments bearing the epitope. The C-terminus fragment resulting from natural proteolytic cleavage and processing of MLL2^FL ^is represented by a 75 kD band in a denaturing gel immunoblot. The uncleaved MLL2^FL ^has a predicted molecular weight of 290 kD. The specificity of the antibody to its target protein, MLL2, was confirmed by using MLL2 specific blocking peptide (Cat # BP300-113, Bethyl labs, Inc., MD, USA) in IHC staining. A part of this blocking peptide represents a portion of the epitope recognized by the anti-MLL2 antibody. Anti-MLL2 antibody (1:30 dilution) was mixed with thirty-fold volumes of the blocking peptide at RT for 0.5 h. This mixture was then added on to a slide with FFPE colon tissue section and incubated for 3 h. The slide was then washed and stained with biotin conjugated secondary antibody, followed by the addition of streptavidin horseradish peroxidase (HRP) conjugate, and visualized by using diaminobenzidine (DAB) as a precipitating enzyme product (Fig. 3D). Other commercially available antibodies against MLL2 were tested, but not found to be suitable for this study.

Immunohistochemical staining for MLL2 in breast and colon tissue sections was performed on the Ventana ES automated IHC instrument (Ventana Medical Systems, Inc., Tucson, AZ) with an indirect biotin/avidin system (Ventana iVIEW detection). Four micron FFPE sections were cut from representative blocks of each case and transferred to glass slides. The tissue sections were de-waxed, rehydrated, and soaked briefly in wash buffer. Unmasking of the MLL2 antigenic determinant sites was not necessary. Immunostaining using anti-MLL2 was carried out at a dilution of 1:30, for 3 h, at 37°C. The specific antibody was localized using a biotin-conjugated secondary antibody (1 h), followed by the addition of a streptavidin horseradish peroxidase (HRP) conjugate. Immunoreactivity was visualized by utilizing DAB as a precipitating enzyme product. Finally, slides were counterstained with hematoxylin. To confirm specificity of primary antibody, negative control slides were run with every batch, using Ventana rabbit polyclonal negative control reagent. Images were acquired using Olympus BX51 (Tokyo, Japan) light microscope with Qimaging digital Camera (Tokyo, Japan) and Qcapture Pro51 software.

#### Immunostaining Interpretation

Cytoplasmic and nuclear MLL2 immunoreactivity was interpreted by a senior pathologist (B.V.S.K), without prior knowledge of the clinicopathology for each case of breast or colon carcinoma. Intensity and distribution were considered by semi quantitative assessment of the staining pattern. The staining intensity was scored as negative, weak (W), moderate (M), or intense (I). The staining distribution was scored as focal (F) (≤ 10%), regional (R) (11 - 50%), or diffuse (D) (>50%). For analysis, intensity and distribution were given equal weight as a multiplicative index, obtained by multiplying intensity by distribution to obtain a total score. Each case was then assigned to one of three categories as follows: cases in which the total score for tumor was less than (T < B; decreased), equal to (T = B), or greater than (T > B; increased) the adjacent benign tissue in the same case.

#### Statistical analysis

Statistical comparisons were carried out using the STATA software (Stata Corporation, College Station, TX). Chi-square test was used to determine significance of the associations between protein expression and prognostic variables. Multivariate analysis, including clinicopathologic parameters and expression of each targeted protein, was performed using the Cox proportional hazards model. The level of significance was set at p < 0.05.

## Abbreviations

**FBS**: Fetal bovine serum; **FFPE**: Formalin fixed paraffin embedded; **H3K4me3**: Histone 3 Lysine 4 tri-methylation; **HB**: Hypotonic buffer; **MLL2**: Myeloid Lymphoid Leukemia 2; **RT-PCR**: Reverse transcription polymerase chain reaction; **IHC**: Immunohistochemistry.

## Competing interests

The authors declare that they have no competing interests.

## Authors' contributions

TGN conceived the study design, conducted in-vitro studies on cell lines, participated in immunohistochemical analysis and drafted the manuscript. BVK conducted histopathological reading of slides and their interpretations. CES performed the immunohistochemical staining and statistical analysis. MBB participated in the in-vitro experiments on cell lines. NG participated in primer design, optimization of immunohistochemical experiments and helped to draft the manuscript. AP participated in optimizing RT-PCR reactions. JSR provided critical comments for improving the manuscript. KTF was involved in study design, revising the manuscript critically for important intellectual content, and gave final approval of the version to be published. All authors read and approved the final manuscript.

## References

[B1] FitzGerald KT Diaz MO MLL2: A new mammalian member of the trx/MLL family of genes Genomics 1999 59 187 192 10.1006/geno.1999.5860 10409430

[B2] Takeda S Chen DY Westergard TD Fisher JK Rubens JA Sasagawa S Kan JT Korsmeyer SJ Cheng EH Hsieh JJ Proteolysis of MLL family proteins is essential for taspase-orchestrated cell cycle progression Genes Dev 2006 20 2397 2409 10.1101/gad.1449406 16951254 PMC1560414

[B3] Demers C Chaturvedi CP Ranish JA Juban G Lai P Morle F Aebersold R Dilworth FJ Groudine M Brand M Activator-mediated recruitment of the MLL2 methyltransferase complex to the beta-globin locus Mol Cell 2007 27 573 584 10.1016/j.molcel.2007.06.022 17707229 PMC2034342

[B4] McKinnell IW Ishibashi J Le GF Punch VG Addicks GC Greenblatt JF Dilworth FJ Rudnicki MA Pax7 activates myogenic genes by recruitment of a histone methyltransferase complex Nat Cell Biol 2008 10 77 84 10.1038/ncb1671 18066051 PMC2739814

[B5] Glaser S Lubitz S Loveland KL Ohbo K Robb L Schwenk F Seibler J Roellig D Kranz A Anastassiadis K Stewart AF The histone 3 lysine 4 methyltransferase, Mll2, is only required briefly in development and spermatogenesis Epigenetics Chromatin 2009 2 5 10.1186/1756-8935-2-5 19348672 PMC2674429

[B6] Glaser S Schaft J Lubitz S Vintersten K van der HF Tufteland KR Aasland R Anastassiadis K Ang SL Stewart AF Multiple epigenetic maintenance factors implicated by the loss of Mll2 in mouse development **2** Development 2006 133 1423 1432 10.1242/dev.02302 16540515

[B7] Lubitz S Glaser S Schaft J Stewart AF Anastassiadis K Increased apoptosis and skewed differentiation in mouse embryonic stem cells lacking the histone methyltransferase Mll2 Mol Biol Cell 2007 18 2356 2366 10.1091/mbc.E06-11-1060 17429066 PMC1877088

[B8] Kelleher FC Fennelly D Rafferty M Common critical pathways in embryogenesis and cancer Acta Oncol 2006 45 375 388 10.1080/02841860600602946 16760173

[B9] Schneider R Bannister AJ Kouzarides T Unsafe SETs: histone lysine methyltransferases and cancer Trends Biochem Sci 2002 27 396 402 10.1016/S0968-0004(02)02141-2 12151224

[B10] Gong W Suzuki K Russell M Riabowol K Function of the ING family of PHD proteins in cancer Int J Biochem Cell Biol 2005 37 1054 1065 10.1016/j.biocel.2004.09.008 15743678

[B11] Sims RJ III Nishioka K Reinberg D Histone lysine methylation: a signature for chromatin function Trends Genet 2003 19 629 639 10.1016/j.tig.2003.09.007 14585615

[B12] Aplan PD Chromosomal translocations involving the MLL gene: molecular mechanisms DNA Repair (Amst) 2006 5 1265 1272 10.1016/j.dnarep.2006.05.034 16797254 PMC1635494

[B13] Huntsman DG Chin SF Muleris M Batley SJ Collins VP Wiedemann LM Aparicio S Caldas C MLL2, the second human homolog of the Drosophila trithorax gene, maps to 9q3. and is amplified in solid tumor cell lines Oncogene 1999 18 7975 7984 10.1038/sj.onc.1203291 10637508

[B14] Rhodes DR Yu J Shanker K Deshpande N Varambally R Ghosh D Barrette T Pandey A Chinnaiyan AM ONCOMINE: a cancer microarray database and integrated data-mining platform Neoplasia 2004 6 1 6 15068665 10.1016/s1476-5586(04)80047-2 PMC1635162

[B15] Neve RM Chin K Fridlyand J Yeh J Baehner FL Fevr T Clark L Bayani N Coppe JP Tong F Speed T Spellman PT DeVries S Lapuk A Wang NJ Kuo WL Stilwell JL Pinkel D Albertson DG Waldman FM McCormick F Dickson RB Johnson MD Lippman M Ethier S Gazdar A Gray JW A collection of breast cancer cell lines for the study of functionally distinct cancer subtypes Cancer Cell 2006 10 515 527 10.1016/j.ccr.2006.10.008 17157791 PMC2730521

[B16] Lacroix M Haibe-Kains B Hennuy B Laes JF Lallemand F Gonze I Cardoso F Piccart M Leclercq G Sotiriou C Gene regulation by phorbol 12-myristate 13-acetate in MCF-7 and MDA-MB-231, two breast cancer cell lines exhibiting highly different phenotypes Oncol Rep 2004 12 701 707 15375488 10.3892/or.12.4.701

[B17] Fogh J Wright WC Loveless JD Absence of HeLa cell contamination in 169 cell lines derived from human tumors J Natl Cancer Inst 1977 58 209 214 833871 10.1093/jnci/58.2.209

[B18] Fukuyama N Jujo S Ito I Shizuma T Myojin K Ishiwata K Nagano M Nakazawa H Mori H Kurozu moromimatsu inhibits tumor growth of Lovo cells in a mouse model in vivo Nutrition 2007 23 81 86 10.1016/j.nut.2006.10.004 17189090

[B19] Langmuir VK McGann JK Buchegger F Sutherland RM 131I-anticarcinoembryonic antigen therapy of LS174T human colon adenocarcinoma spheroids Cancer Res 1989 49 3401 3406 2720694

[B20] Semple TU Quinn LA Woods LK Moore GE Tumor and lymphoid cell lines from a patient with carcinoma of the colon for a cytotoxicity model Cancer Res 1978 38 1345 1355 565251

[B21] Weber TK Steele G Summerhayes IC Differential pp60c-src activity in well and poorly differentiated human colon carcinomas and cell lines J Clin Invest 1992 90 815 821 10.1172/JCI115956 1381724 PMC329935

[B22] Lee S Lee DK Dou Y Lee J Lee B Kwak E Kong YY Lee SK Roeder RG Lee JW Coactivator as a target gene specificity determinant for histone H3 lysine 4 methyltransferases Proc Natl Acad Sci USA 2006 103 15392 15397 10.1073/pnas.0607313103 17021013 PMC1622834

[B23] Lee S Lee J Lee SK Lee JW Activating signal cointegrator-2 is an essential adaptor to recruit histone H3 lysine 4 methyltransferases MLL3 and MLL4 to the liver X receptors Mol Endocrinol 2008 22 1312 1319 10.1210/me.2008-0012 18372346 PMC2422828

[B24] Draffin JE McFarlane S Hill A Johnston PG Waugh DJ CD44 potentiates the adherence of metastatic prostate and breast cancer cells to bone marrow endothelial cells Cancer Res 2004 64 5702 5711 10.1158/0008-5472.CAN-04-0389 15313910

[B25] Burdall SE Hanby AM Lansdown MR Speirs V Breast cancer cell lines: friend or foe? Breast Cancer Res 2003 5 89 95 10.1186/bcr577 12631387 PMC154155

[B26] Song JJ Kingston RE WDR5 interacts with mixed lineage leukemia (MLL) protein via the histone H3-binding pocket J Biol Chem 2008 283 35258 35264 10.1074/jbc.M806900200 18840606 PMC2596411

[B27] Hughes CM Rozenblatt-Rosen O Milne TA Copeland TD Levine SS Lee JC Hayes DN Shanmugam KS Bhattacharjee A Biondi CA Kay GF Hayward NK Hess JL Meyerson M Menin associates with a trithorax family histone methyltransferase complex and with the hoxc8 locus Mol Cell 2004 13 587 597 10.1016/S1097-2765(04)00081-4 14992727

[B28] Li M Brooks CL Wu-Baer F Chen D Baer R Gu W Mono- versus polyubiquitination: differential control of p53 fate by Mdm2 Science 2003 302 1972 1975 10.1126/science.1091362 14671306

[B29] Elston CW Ellis IO Pathological prognostic factors in breast cancer. I. The value of histological grade in breast cancer: experience from a large study with long-term follow-up Histopathology 1991 19 403 410 10.1111/j.1365-2559.1991.tb00229.x 1757079

[B30] American Joint Committee Manual for staging of cancer 1998 Philadelphia: J.B. Lippincott

[B31] Dukes CE Histological grading of rectal cancer Proceedings of the royal society of medicine 1937 30 371 376 19990989 10.1177/003591573703000412 PMC2076469

